# In pursuit of an accurate spatial and temporal model of biomolecules at the atomistic level: a perspective on computer simulation

**DOI:** 10.1107/S1399004714026777

**Published:** 2015-01-01

**Authors:** Alan Gray, Oliver G. Harlen, Sarah A. Harris, Syma Khalid, Yuk Ming Leung, Richard Lonsdale, Adrian J. Mulholland, Arwen R. Pearson, Daniel J. Read, Robin A. Richardson

**Affiliations:** a The Edinburgh Parallel Computing Centre, The University of Edinburgh, Edinburgh EH9 3JZ, Scotland; b School of Mathematics, University of Leeds, Leeds LS2 9JT, England; c School of Physics and Astronomy, University of Leeds, Leeds LS2 9JT, England; d Astbury Centre for Structural Molecular Biology, University of Leeds, Leeds LS2 9JT, England; e Faculty of Natural and Environmental Sciences, University of Southampton, Southampton SO17 1BJ, England; f Max-Planck-Institut für Kohlenforschung, Kaiser-Wilhelm-Platz 1, 45470 Mülheim an der Ruhr, Germany; g Department of Chemistry, Philipps-Universität Marburg, Hans-Meerwein Strasse, 35032 Marburg, Germany; h Centre for Computational Chemistry, University of Bristol, Bristol BS8 1TS, England; i Hamburg Centre for Ultrafast Imaging, University of Hamburg, Hamburg, Germany

**Keywords:** biomolecular simulation, computational techniques

## Abstract

Despite huge advances in the computational techniques available for simulating biomolecules at the quantum-mechanical, atomistic and coarse-grained levels, there is still a widespread perception amongst the experimental community that these calculations are highly specialist and are not generally applicable by researchers outside the theoretical community. In this article, the successes and limitations of biomolecular simulation and the further developments that are likely in the near future are discussed. A brief overview is also provided of the experimental biophysical methods that are commonly used to probe biomolecular structure and dynamics, and the accuracy of the information that can be obtained from each is compared with that from modelling. It is concluded that progress towards an accurate spatial and temporal model of biomacromolecules requires a combination of all of these biophysical techniques, both experimental and computational.

## Abstract 2

The current computational techniques available for biomolecular simulation are described, and the successes and limitations of each with reference to the experimental biophysical methods that they complement are presented.

## 1. Introduction   

High-throughput sequencing, protein production and crystallization, and developments in X-ray crystallography, small-angle and wide-angle X-ray scattering (SAXS/WAXS), electron microscopy (EM), mass spectrometry and nuclear magnetic resonance (NMR), combined with modern data-storage capacities, have enormously increased the quantity of biological information available in structural, proteomics and genomics databases. However, without an equivalent investment in understanding and interpretation in terms of biological function, our ability to use this information is limited. Computer modelling to enhance our understanding and guide further experimental characterization is essential, since the quantity and complexity of biological data is immense and continues to grow.

Improved understanding of biomolecular interactions and their consequences through computer modelling is not just of fundamental interest, but is also of benefit to applied science. Structural molecular biology has provided the key concepts underlying rational drug design, which enables the use of molecular design to target an active site or binding pocket of a known structure. The desired outcome is usually a molecule that specifically recognizes the correct binding site with sufficient affinity to outcompete the natural substrate. However, it is still not always possible to accurately predict the binding constant simply from knowledge of the structures of the individual binding partners because of a lack of a fully quantitative understanding of the relationship between molecular structure and thermodynamics. In spite of some successes, it can also still be difficult to interpret calorimetric data, for example gained by techniques such as isothermal titration calorimetry (ITC), in terms of atomistic structure alone, and this has proven troublesome to the pharmaceutical industry, who would ideally use the insight provided by these techniques to enhance drug development (Chaires, 2008 ▶).

**Figure 1:**
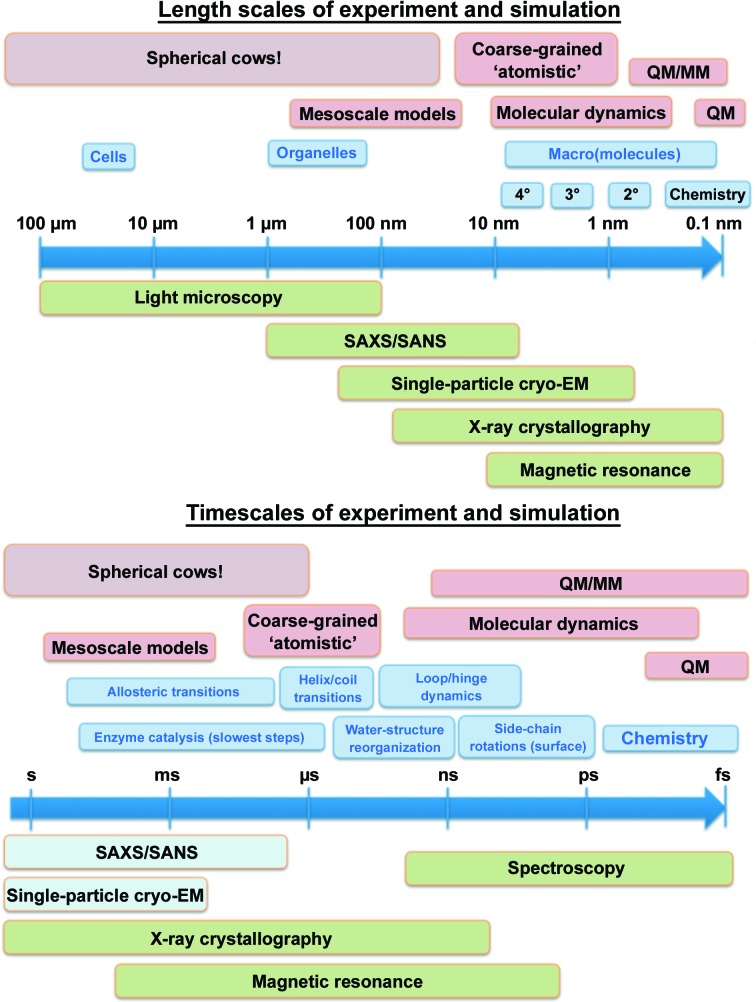
A comparison of the molecular-biophysics length scales and timescales accessible to simulation and experiment. The term ‘spherical cows’ refers to approximate models that provide an abstract representation of a physical system; such models are useful because of the simplicity of the calculations (*e.g.* volumes or surface areas of a herd can be easily estimated by assuming that cows are spherical and assigning a radius) and the general nature of the model (the volume of a goat herd can easily be compared).

A purely structural description has proven to be insufficient for a complete understanding of biological function because biomolecules are conformationally flexible objects. They change shape significantly owing to thermal fluctuations when they are at 300 K, and site-specific recognition can involve large conformational changes as the biomolecules adapt their flexible conformations to maximize favourable interactions. In the case of allosteric interactions, these conformational changes can give rise to information transfer through both changes in shape and flexibility (*e.g.* entropy); dynamic information transfer has, for example, been demonstrated theoretically by Rodgers *et al.* (2013 ▶) and experimentally by Tzeng & Kalodimos (2012 ▶). However, directly visualizing these structural rearrangements and changes in biomolecular flexibility remains challenging. Mesostable states can be visualized by X-ray crystallography and gross structural rearrangements are observable by EM and SAXS/WAXS, but a true time-resolved atomistic description of the functionally related conformational rearrangements remains elusive for most systems. NMR provides detailed information on sample dynamics, but remains limited by molecule size and the need for isotopic labelling. Spectroscopic methods [electron paramagnetic resonance (EPR), Förster resonance energy transfer (FRET) and optical and vibrational spectroscopies] provide detailed information on either very local structural rearrangements or long-range distance changes, but the challenge here lies in linking this sparse information to a complete description of the molecular system (Fig. 1 ▶). In response to this, biomolecular simulation has developed algorithms which calculate the thermal motion of biomolecules, in the hope that this will offer a theoretical bridge between thermodynamic measurements, sparse distance information and atomistic structure. Although promising, these simulation methods have not replaced experiments owing to the approximations that are currently needed for the calculations to be tractable. However, in common with experiments, but unlike mathematical modelling (for example using a ‘spherical cows’ approach; see Fig. 1 ▶), the value of computer simulations improves with improving technology.

In this article, we discuss the successes and limitations of biomolecular simulations and the further improvements that are likely in the near future. We also provide a brief overview of the experimental biophysical methods commonly used to probe biomolecular structure and dynamics and compare the accuracy of the information that can be obtained from each with that from simulation. We conclude that progress towards an accurate spatial and temporal model of biomacro­molecules requires an integrated approach that uses a combination of all of these biophysical techniques, both experimental and theor­etical (Zhao & Schuck, 2015 ▶).

## 2. The ‘resolution’ of biophysical methods and comparison with simulation   

In experimental biophysics, the term resolution refers to the spatial and temporal dimensions accessible to a technique. In addition, it should be noted that most biophysical experiments are ensemble measurements that study the behaviour of a population of macromolecules. Owing to the conformational heterogeneity of macromolecules, this ensemble averaging results in a loss of both spatial and temporal resolution. The exceptions are single-molecule experiments. However, although these are producing results about the physical properties of macromolecules (atomic force microscopy), reaction kinetics (single-enzyme kinetics) and subcellular localization (super-resolution microscopy), they do not yet provide atomic resolution information about the macromolecular structure.

We can broadly divide experimental biophysical techniques into three classes. The first includes those techniques that are able to provide both a high-resolution structural and potentially temporal description of the entire macromolecule (*i.e.* X-ray crystallography and EM). The second class comprises techniques that provide sparse spatial information [*i.e.* EPR, FRET, ITC, surface plasmon resonance (SPR), optical/vibrational spectroscopy and single-molecule biophysics] that can take the form of direct distance measurements, kinetics, binding interaction data or very local spatial information. These data, whilst sparse, can be of extremely high temporal and spatial resolution. The third class includes those techniques which can provide low-resolution structural information on the entire macromolecule as well as information on dynamics [SAXS/small-angle neutron scattering (SANS)/WAXS, mass spectrometry, hydrogen/deuterium exchange (HDX) and footprinting]. NMR is a special and extremely versatile case as, depending on the exact experiment carried out, it could be classified into any of the three classes. Indeed, as long as an appropriate pulse sequence and sample-labelling methodology can be devised, NMR can be used to probe the structure and dynamics of the system of interest. A major advantage of NMR is that the requirement for paramagnetic nuclei for detection means that NMR can probe the structure and dynamics of the molecule of interest in extremely complex backgrounds such as crowded macromolecular environments (Cabrita *et al.*, 2010 ▶).

In spite of the wealth of biophysics techniques available, in the main structural molecular biology has made progress by using experimental design which simplifies highly complex biological systems. For example, binding-affinity measurements and ITC are performed in dilute solution under conditions where only the binding partners of interest are included, under the assumption that the cellular environment is not significant. However, fundamental processes such as protein folding, which are commonly studied in isolation, are known to be influenced by the presence of the ribosome and/or chaperones. X-ray crystallography requires a homogeneous crystalline environment which may more closely reflect the crowded environment of the cell, but does not reflect its heterogeneity. Indeed, the effects of crowding are clearly important for *in vivo* protein function (Ellis, 2001 ▶).

Model building and approximation are already endemic in the biosciences, as it has been necessary to take a reductionist approach when designing experiments to overcome the complexity inherent in molecular biology; consequently, nearly all *in vitro* experiments are an abstraction of the real *in vivo* system. Computer simulations provide a different, but no less valid, and often more detailed, technique for model building. Currently, most simulations begin from data provided by experimentalists and this places a large responsibility on both the experimenter and theoretician to understand the capabilities, limitations and assumptions of each, as has been previously discussed (Coveney & Fowler, 2005 ▶).

## 3. Computer simulations of biomolecules   

Computer simulations of biomolecules aim to integrate high-accuracy physical models with efficient parallel computer algorithms running on the best available supercomputing hardware, which often requires infrastructure at the national level, such as the UK supercomputer ARCHER (hardware will be discussed in §[Sec sec4]4). Simulation algorithms are continuously updated to exploit advances in computing architectures, such as graphics processing units (GPUs), and atomistic molecular-dynamics (MD) simulations have been considered to be sufficiently useful to society that Shaw Research have designed a special-purpose parallel architecture for fast MD, known as Anton, which has been reported to run simulations two orders of magnitude faster than conventional resources (Dror *et al.*, 2012 ▶). Since a more accurate physical description nearly always entails higher computational expense, including fewer spatial details usually allows longer timescales and length scales to be explored. Molecular models at the quantum and atomistic levels require that the atomistic structure of the biomolecule is known. Either the experimental coordinates are downloaded from the Protein Data Bank (PDB), or the structure has to be predicted by homology modelling. Recent developments in high-resolution cryo-EM promise to provide a new source of high-resolution structural data (Brown *et al.*, 2015 ▶; Lučič *et al.*, 2013 ▶). Coarse-grained simulation methods do not necessarily suffer from this restriction, and can take advantage of the growing Electron Microscopy Data Bank (EMDB). The aim is then to use computation to calculate quantities that are unobtainable from experiments alone, such as the magnitude of conformational changes owing to thermal motion. While robust validation is always required when predictions go beyond what is experimentally accessible, the range of applicability of the models is usually well known and the limitations understood. The precise validation required and the nature of these limitations will be specific to the biological question being addressed, and requires careful thought on behalf of the simulator. An example, however, would be good agreement between the time-averaged structure of a protein calculated from an MD simulation and the X-ray structure used as input to the simulations (Rueda *et al.*, 2007 ▶).

The following section aims to provide a flavour of the wealth of computational studies that have been performed for biomolecules at each particular level of accuracy using specific examples, but is by no means intended to be exhaustive. Consequently, we refer the curious reader to the many reviews cited for a more detailed account of a particular biomolecular simulation technique. We also encourage researchers who wish to perform their own simulations to engage with the comprehensive series of tutorials and workshops provided by the websites of biomolecular simulation software codes such as *GROMACS* (http://www.gromacs.org/Documentation/Tutorials) or *AMBER* (http://ambermd.org/tutorials/), and invite them to attend the practical workshops run by CCP5 (http://www.ccp5.ac.uk/events/) and by CCP-BioSim (http://www.ccpbiosim.ac.uk/workshops).

### 3.1. Quantum-mechanical calculations   

Whilst reactions involving the reconfiguration of covalent bonds are ubiquitous in biochemistry, representing the distribution of electrons within biomolecules is extremely computationally expensive. Nevertheless, quantum-mechanical (QM) calculations have been used, for example, to understand the catalytic ability of enzymes, to predict how key mutations may affect catalytic efficiency and to explain the high selectivity of enzyme-catalysed reactions. They have been used to reveal the structures of short-lived species such as transition states, which can then be used as templates for the design of enzyme inhibitors for biotechnological or medicinal applications (Lonsdale *et al.*, 2012*a* ▶).

A typical quantum-chemical calculation solves the Schrödinger equation to determine the energy and electronic configuration of the valence electrons associated with a particular set of coordinates for the atomic centres. Determining the electronic structure shows to what extent a given pair of atoms are covalently bonded, where the electrons are distributed and where the biomolecule is polar or apolar, and can provide the empirical parameters required for classical atomistic simulations. When applied to enzyme catalysis, QM calculations have been used to understand how the enzyme lowers the activation-energy barrier of the chemical reaction by perturbing the electronic structure of the transition state. Short-lived species such as transition states are hard to isolate experimentally, hence calculations are used to provide information that would otherwise be inaccessible. In complex biochemical reactions, the detailed mechanism of the catalysed reaction may not be well understood, and in this situation quantum-chemical calculations can test and compare hypotheses *in silico*. When combined with geometry-optimization algorithms, which adjust the relative positions of the atomic nuclei in conjunction with the molecular orbitals, quantum-chemistry calculations can show how the shape and electronic structure of biomolecular fragments change along a reaction pathway on the sub-ångström length scale. Many QM methods are too computationally intensive to permit molecular-dynamics (MD) simulations and instead are used to calculate potential energy profiles, from which one may deduce the pathway taken during a chemical reaction by calculating the lowest energy route across a potential energy landscape. Relative reaction rates, for example when residues in an enzyme active site are mutated, can then be predicted by comparing the heights of the energy barriers between the reactants and products. Reaction rates can also be compared between different substrates. Also, by comparing the relative energy barriers to different proposed mechanisms, the most likely mechanism can be deduced (*i.e.* that with the lowest barrier).

The bottlenecks in quantum-chemical calculations are usually the matrix-diagonalization operations to obtain the wavefunctions of the molecular orbitals occupied by the valence electrons in the molecule, which are both slow and memory intensive. The so-called post-Hartree–Fock *ab initio* methods are the most accurate QM methods, and examples of these include Møller–Plesset perturbation theory (MP2) and coupled-cluster theory. These methods can be used to study model reactions that involve a relatively small number of atoms (∼20) to a high degree of accuracy, *e.g.* for gas-phase bimolecular reactions or for small cluster models of enzyme active sites. For reactions involving significantly more atoms, *e.g.* for enzymatic reactions with multiple residues participating in the mechanism, these methods are usually not feasible for routine application. The cheapest QM methods are the semi-empirical methods (*e.g.* AM1 and PM3), the name arising from the fact that the more expensive parts of the QM calculation are approximated by a set of parameterized functions that are based on empirical data. These are used for calculations requiring a large number of QM atoms (∼100 atoms) and for calculating free-energy barriers. However, semi-empirical methods have limited applicability because they are parameterized for a finite set of reactions and are not derived from first principles. Whilst they have been shown to provide useful insight for reactions that are similar to those that they have been parameterized for, their accuracy is less good for reactions which deviate too far from the parameterization set. Density-functional theory (DFT), which is intermediate in complexity between semi-empirical and *ab initio* methods, provides a good compromise between accuracy and computational cost. However, DFT calculations generally do not include dispersion (*e.g.* van der Waals) interactions, so for biological molecules in which such forces play an important role in stabilizing folded conformations these need to be added empirically (Lonsdale *et al.*, 2010 ▶, 2012*b* ▶). There are various types of density functional which differ in the way in which electron exchange and correlation is mathematically represented. One of the most popular functionals is B3LYP, which has been used successfully to calculate molecular geometries and energies reasonably accurately compared with more computationally intensive methods. It is known as a hybrid functional, because the electron exchange is calculated using a linear combination of three different methods, and their relative contributions have been parameterized to fit to experimental data.

For large biomolecules where the active site forms a small section of the entire protein, hybrid quantum-mechanical/molecular-mechanical (QM/MM) methods are used (van der Kamp & Mulholland, 2013 ▶). QM/MM calculations use classical molecular mechanics (MM) to represent the atoms that do not take part in the chemical reaction, but treat the active-site quantum mechanically. The classically treated atoms within the model affect the energies of the chemically reacting species firstly through the presence of the MM region placing a restriction on the movement of the QM region during minimization, *i.e.* the MM atoms hold the QM region in position, and secondly through long-range electrostatic interactions, although only the more accurate QM/MM methods allow the MM region to polarize the QM region. In addition, the protein as a whole may routinely undergo large conformational changes during its thermal motion, so an ensemble of structures (at least five) is considered in state-of-the-art QM/MM models to ensure that the changing shape of the active site is included in the potential energy surface calculations. This ensemble of structures is usually obtained from an MD simulation of the enzyme–substrate complex (MD simulations are discussed in more detail in §[Sec sec3.2]3.2).

**Figure 2:**
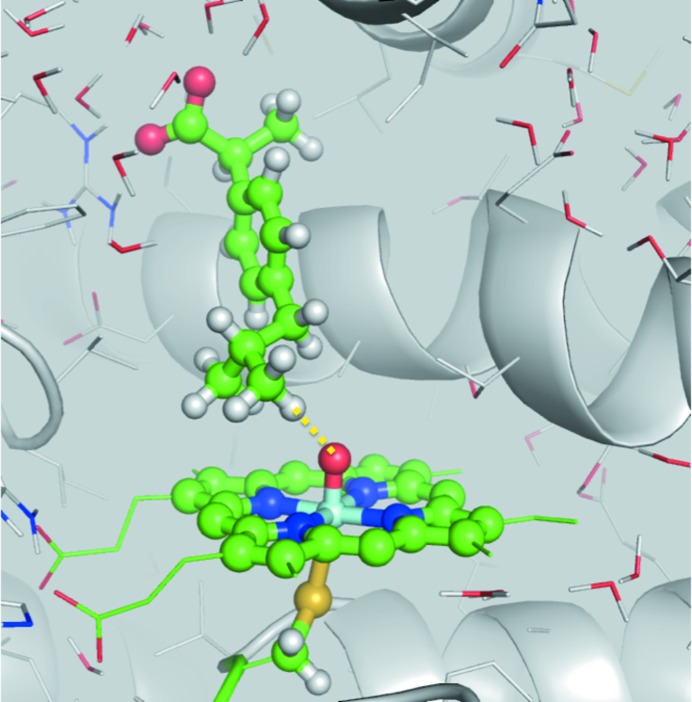
The transition state for the hydroxylation of *S*-ibuprofen at C3 in the human drug-metabolizing cytochrome P450 2C9 (Lonsdale *et al.*, 2013 ▶). The H atom at C3 undergoes abstraction by the ferryl O atom of the porphyrin (shown as a yellow dashed line). Knowledge of the mechanism and transition states for reactions such as these can be useful in the design of new pharmaceutical compounds with desired metabolic properties. The reaction was modelled with QM/MM (using the *QoMMMa* program; Harvey, 2004 ▶) using multiple starting structures taken from MD simulations (performed using *CHARMM*; Brooks *et al.*, 1983 ▶) and the CHARMM27 force field (MacKerell *et al.*, 2000 ▶). The QM region is shown in ball-and-stick representation.

Knowledge of the factors that determine the reactivity and selectivity of drug metabolism is useful in the design of new pharmaceutical compounds that do not result in the formation of toxic metabolites (Lonsdale & Mulholland, 2014 ▶). An example application of QM/MM methods for studying drug metabolism focused on the regioselectivity of hydroxylation of drug molecules by the human cytochrome P450 2C9 enzyme. The QM region was modelled using the B3LYP density functional (with an empirical dispersion correction), and the rest of the enzyme and the solvent were represented using the CHARMM27 force field, as shown in Fig. 2 ▶ and Supplementary Movies S1 and S2 (Lonsdale *et al.*, 2013 ▶). The calculations showed that relatively small changes in substrate orientation relative to active-site residues can have a dramatic effect on the heights of relevant energy barriers. The reactive species in P450s is a highly reactive iron(IV)–oxo species (called Compound I) which is difficult to isolate experimentally and has a complex electronic structure (Bathelt *et al.*, 2005 ▶). Calculating the energy barriers to the reaction at different sites of the reacting molecules found that the lowest barriers in two out of three cases corresponded to the oxidation sites observed experimentally, which provided good evidence that QM/MM calculations can be used to reliably predict the site of metabolism for drugs in P450 enzymes. These calculations also revealed that the mechanism underlying the selectivity of P450 enzymes is a combination of factors involving the orientation of the substrate in the active site and the differing reactivities of chemical sites on the substrate. In common with many enzymes, the human cytochromes P450 are membrane-bound; however, all known crystal structures to date have been obtained for solubilized forms in the absence of membrane. In a recent study, a membrane-bound model of the human CYP3A4 isoform was constructed using a combination of atomistic and coarse-grained molecular-dynamics simulations (Lonsdale *et al.*, 2014 ▶). QM/MM (B3LYP-D/CHARMM27) reaction profiles were calculated both from membrane-bound and soluble forms of the enzyme. The calculations revealed that the reactivity of the enzyme is similar between the two forms; however, important differences were observed between the substrate entrance and exit pathways. It is hoped that the framework outlined in this study can be applied to the study of other membrane-bound enzymes.

In principle, QM/MM methods should be applicable to all enzymes, yet whilst many enzymes have been studied using such methods (van der Kamp & Mulholland, 2013 ▶; Ranaghan & Mulholland, 2009 ▶), these calculations are not yet routine. The choice of an appropriate QM method depends on the system and the type of problem to be addressed. Also, the size of the QM region and other practical aspects are important and should be thoroughly tested for each new application (Lonsdale *et al.*, 2012*a* ▶). The classical MM region is treated using invariant point charges and (unless a polarizable MM model is used) cannot be polarized by changes in charge on the QM atoms. This can be overcome by increasing the size of the QM region to incorporate more of the surrounding atoms, but this increases the computational expense. Conformational sampling is also an important issue, and reaction profiles should be calculated starting from different conformations of the enzyme (*i.e.* using different structures from an MD simulation), which additionally increases the computational cost because it requires each calculation to be performed multiple times.

### 3.2. Classical atomistic molecular dynamics (MD)   

Atomistic molecular-dynamics (MD) simulations have famously been described as providing a ‘computational microscope for molecular biology’ (Dror *et al.*, 2012 ▶) because simulations viewed on a computer screen can create the impression that the biomolecule has been magnified to sufficient dimensions that it is being visualized with optical microscopy. MD provides a trajectory of a biomolecule which shows how it changes shape as it undergoes thermal fluctuations at 300 K, as shown in Supplementary Movie S3. The MD algorithm treats every atom within the protein as a classical ball connected by covalent bonds, which are represented as perfect harmonic springs and which therefore cannot break. Nonbonded atom pairs interact through van der Waals (or dispersion) interactions and electrostatics. Each atom in the system is assigned a set of empirical parameters designed to impart chemical specificity, which are known as the MD force-field parameters. For example, each atom carries a partial charge depending upon its electronic properties and its environment. MD force-field parameters are derived from a combination of experimental data (such as vibrational spectroscopy of small molecules) and QM calculations on tractable molecular fragments, and are constantly being iteratively debated, checked and improved. The solvent environment is generally also represented at the atomistic level. Consequently, calculations involving proteins in membranes are more computationally expensive owing to the need to simulate the lipid bilayer. The change in position of every atom in response to the force that it experiences from all of the others is calculated over a very short numerical integration timestep (typically 2 fs) using Newtonian mechanics. The bottleneck in MD arises from the enormous computational expense of calculating the net force on every particle from all other particles in the system at each short timestep interval. However, if the timestep is too long then the energy will progressively inflate and the simulation will become numerically unstable. Consequently, even with parallel computing, MD simulations exploring timescales of tens of microseconds are currently considered to be state of the art, although timescales of milliseconds have been achieved using specialized resources (Shaw *et al.*, 2010 ▶).

Atomistic molecular-dynamics simulation is arguably the most mature biomolecular modelling technique. Recent research highlights from this field include the MoDEL simulation database, which provides the biomolecular sciences community with a database containing in excess of 1700 protein trajectories obtained by state-of-the-art MD simulations which can be freely downloaded (Meyer *et al.*, 2010 ▶), insights into protein-folding mechanisms for fast folders (Lindorff-Larsen *et al.*, 2011 ▶), atomistic information on the structure and dynamics of 100 nm cages constructed from self-assembling coiled-coil peptides to complement scanning electron-microscopy data (Fletcher *et al.*, 2013 ▶) and simulations of the entire tubular HIV-1 capsid assembly based on cryo-electron tomography (Zhao *et al.*, 2013 ▶). There is an established software infrastructure used by a global simulation community, such as the MD codes *NAMD* (Phillips *et al.*, 2005 ▶), *GROMACS* (Pronk *et al.*, 2013 ▶), *CHARMM* (Brooks *et al.*, 1983 ▶) and *AMBER* (Case *et al.*, 2014 ▶), and accompanying visualization tools such as *VMD* (Humphrey *et al.*, 1996 ▶), *Chimera* (Pettersen *et al.*, 2004 ▶) and *PyMOL* (Schrödinger). MD computer programs have been optimized for parallel performance on national resources and new architectures such as GPUs, and are generally free to academics. While a user can chose between AMBER or CHARMM force-field parameters, many simulators routinely compare the results from several force fields as there are no set rules for choosing one over the other, although the differences are usually small (Rueda *et al.*, 2007 ▶). Multiple replicate simulations (typically at least three, but more than ten are now common) are also required because thermal effects can be so significant at 300 K that a chance event observed in a simulation can be mistaken for an important mechanistic result.

While the Nobel Prize for Chemistry in 2013 was awarded for the development of multiscale models of complex chemical systems, and in spite of the fact that the first successful biomolecular simulation was reported in 1977 (McCammon *et al.*, 1977 ▶), it is disappointing that we are still unable to routinely provide an accurate prediction of biomolecular affinities (which are governed by the change in free energy when biomolecules associate) using atomistic simulation, even for small molecules. However, the physical attributes that allow proteins to perform such extraordinary functions *in vivo* also make their interactions very challenging to describe quantitatively. Many proteins act as switches or participate in signalling cascades. Therefore, biomolecular affinities are often modulated through allosteric interactions with other molecules or by environmental perturbations, such as changes in pH, temperature or salt concentration. To be switchable, free-energy differences must be delicately balanced, which implies that they are modest in magnitude compared with the thermal energy. This is achieved thermodynamically firstly because all biomolecular interactions are mediated by the solvent; binding partners need to displace a layer of bound solvent before the interaction can proceed. Assuming that these solvent interactions can be overcome, the remaining favourable interaction energy that drives molecular association is generally (but not always) offset by unfavourable changes in entropy, which occur because the conformational flexibility of any molecule tightly bound within a complex is usually reduced relative to its unbound state. Consequently, the overall free-energy change that drives molecular recognition involves a number of large but compensating terms that are opposite in sign, with the result that even a small error in the calculation of any one component leads to a large error in the overall value predicted by theory.

The importance of dynamics and entropy in biomolecular function is particular challenging for computation, because calculation of the entropy requires a full exploration of the conformational space of the biomolecule. In response to this, the community has developed a plethora of innovative computational and algorithmic techniques designed to provide a more efficient exploration of conformational space (Zuckerman, 2011 ▶). Examples include replica exchange, which uses elevated temperatures to force the biomolecule to move more rapidly across its free-energy landscape, and metadynamics or conformational flooding, which impose restraints upon a biomolecule that place a bias against revisiting areas of conformational space that the simulator has already observed, as described by Zuckerman (2011 ▶). While much progress has been made using these techniques, especially in the area of drug design (see part VI of Baron, 2012 ▶), they are certainly not routine. More sophisticated algorithms often provide results that are more difficult to interpret, and care must be taken that any additional artefacts that are introduced are not hidden behind this extra layer of complexity.

### 3.3. Coarse-grained biomolecular simulation   

**Figure 3:**
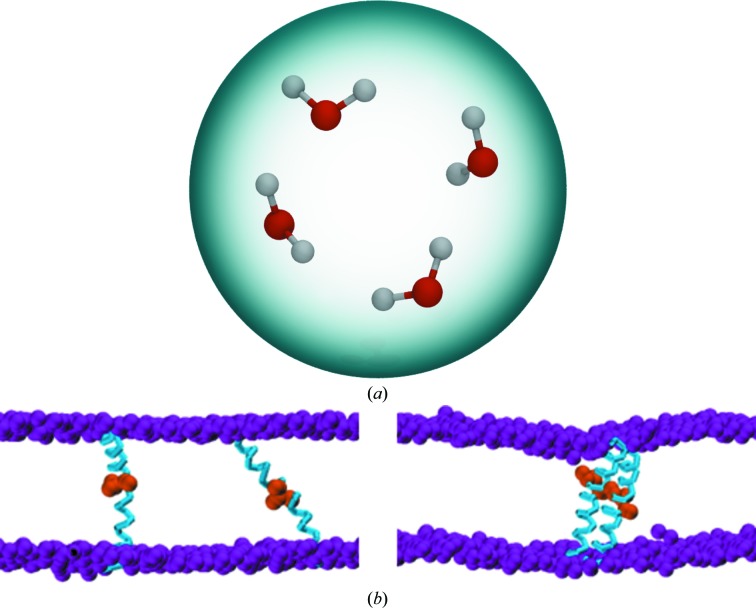
Coarse-grained biomolecular simulations. (*a*) A schematic of the water model in which four atomistic water molecules are lumped together in a single particle (indicated by the grey sphere). (*b*) Initial (left) and final (right) positions observed during the dimerization of fukutin. The protein backbones are shown in cyan, the S residues of the important T*XX*SS motif are shown in orange space-filling format, the phosphate groups of the lipids are shown in purple space-filling format, and the remainder of the lipid molecules and the waters have been omitted for clarity.

Atomistic molecular-dynamics simulations are computationally very demanding; each atom is considered to be a single particle, which results in many interaction sites. The greater the number of interaction sites, the slower the simulation. These simulations can become prohibitively slow for studying processes such as the self-assembly of lipid bilayers and protein-oligomerization events. An alternative approach is to use coarse-grained models (for a recent review, see Tozzini, 2010 ▶). In such models, a group of heavy (non-H) atoms are combined together into a single, larger particle, thereby reducing the number of interaction sites. A coarse-grained simulation can thus access longer timescales and length scales than is possible by atomistic simulations, albeit at the cost of the atomistic detail. The speed-up in the sampling of phase space achieved by CG force fields for biomolecular simulation can vary between five and ten times faster (Marrink *et al.*, 2004 ▶) to 15–200 times faster (Orsi & Essex, 2011 ▶). The simulation speed-up is a result of the reduced system size, but also the ‘softer’ potentials used to describe the interactions within the particles, which result in smoother energy landscapes compared with atomistic simulations and enable longer integration timesteps to be used. While many coarse-grained models have been reported and are widely used within the wider biomolecular simulation community, arguably the most popular is currently the MARTINI force field (Marrink *et al.*, 2004 ▶; Marrink & Tieleman, 2013 ▶). In general, four heavy atoms are lumped together into a single particle in MARTINI, which gives rise to the representation of water shown in Fig. 3 ▶(*a*). MARTINI is usually implemented within the *GROMACS* simulation package; for examples, see Bond *et al.* (2007 ▶) and Scott *et al.* (2008 ▶).

An example of the use of coarse-grained biomolecular simulation to study systems that would be computationally inaccessible to atomistic calculations is provided by Supplementary Movie S4, which shows the self-assembly of lipids around an outer membrane protein (Bond & Sansom, 2006 ▶). Supplementary Movie S5 and Fig. 3 ▶(*b*) show a recent study of the membrane protein fukutin (Marius *et al.*, 2012 ▶; the lipid membrane has been omitted for clarity). Fukutin resides in the endoplasmic reticulum or the Golgi apparatus within the cell. Its localization is thought to be mediated by the inter­action of its N-terminal transmembrane domain with the surrounding mem­branes. Experimental work has shown that this domain exists as a dimer within the lipid bilayers; however, the process of dimerization, the structure of the dimers and the localization within the membranes are difficult to probe using experimental methods alone. Coarse-grained MD simulations predict steps in the dimerization process, in which a T*XX*SS motif is crucial in holding the dimer together. Fig. 3 ▶(*b*) (left) shows the initial positions of the N-terminal transmembrane domains of two fukutin proteins compared with their dimerized state after 2 µs of coarse-grained MD simulation (Fig. 3 ▶ *b*, right).

Much of biomolecular simulation has been inspired by the wealth of structures available in the PDB, and has therefore been focused on providing theoretical methods that use this information as direct input to computations. However, progress in lower resolution methods, such as cryo-electron microscopy and cryo-tomography, is leading to a rapid growth in the number of structures available in the EMDB. In response, biomolecular simulators have designed simulation techniques that take advantage of these new experimental data (Kim *et al.*, 2011 ▶). While the PDB provides atomistic structures, the EMDB provides lower resolution volumetric information illustrating the overall shape of biomolecules, biomolecular complexes or supermolecular structures. Therefore, one strategy for simulating EMDB maps is to use a continuum representation in which no atoms or particles are present at all. Such an approach is common at the macroscopic level. Finite-element analysis (FEA) is used ubiquitously for computer-aided design within structural engineering applications, but does not take thermal noise into account.

**Figure 4:**
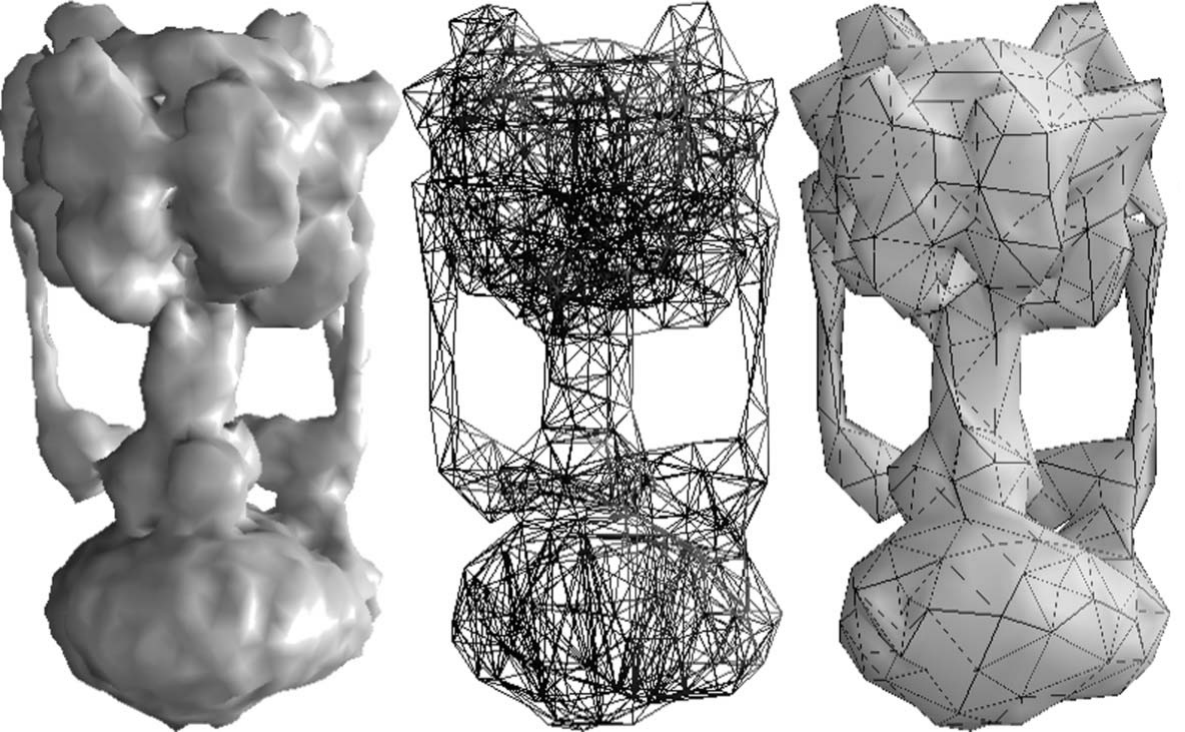
From left to right, this figure shows the EMDB density map of the *Thermus thermophilus* A-­ATPase, the corresponding FFEA mesh and the ‘solid’ version of this mesh (Richardson *et al.*, 2014 ▶).

Fluctuating finite-element analysis (FFEA) is a generalization of FEA to objects which are sufficiently small that thermal fluctuations are non-negligible in magnitude (Oliver *et al.*, 2013 ▶). In FFEA, the complex shape of the protein is represented by a three-dimensional mesh of elements, with the most convenient element shape being the tetrahedron, as shown for the rotary ATPase motor in Fig. 4 ▶. This mesh is then parameterized with continuum material parameters such as the density of the protein, its Young’s modulus and the viscosities of the biomacromolecule and its solvent environment. These material parameters should describe the cumulative effect at the continuum level of all of the local atomic inter­actions. Once these parameters have been defined, the trajectory describing the changing shape of the protein owing to thermal fluctuations can be calculated by iteratively integrating Newton’s equations of motion over short timesteps and moving each node of the mesh accordingly, as shown for the rotary ATPase motor in Supplementary Movie S6 (Richardson *et al.*, 2014 ▶). The calculation is analogous to conventional molecular dynamics (MD), but the forces on each node within the mesh are derived from continuum mechanics equations rather than a pairwise particle-based force field. Since it operates in the continuum limit it has no upper length scale, and it is sufficiently coarse-grained to enable simulations of very large protein structures to be performed for long (*e.g.* microsecond) timescales. As long as the necessary force-field parameters can be obtained, FFEA can include intermolecular forces between biomolecules (such as van der Waals and electrostatics interactions) and it is also possible to simulate collections of interacting proteins, protein association and disassociation or proteins immersed in complex subcellular environments.

An alternative approach for studying very large proteins and protein complexes improves computational efficiency by simplifying the mathematical equations rather than the biomolecules themselves. Gaussian and elastic network models (ENMs) are a widely used class of structure-based coarse-grained models for proteins which represent the native structure of the protein as an elastic body comprised of a set of nodes connected by springs (Noid, 2013 ▶). Using simple springs to represent all of the intermolecular interactions within the protein enables the equations of motion to be solved exactly by a procedure known as normal-mode analysis, without the need to run a simulation or obtain a dynamical trajectory. The calculation provides a set of structural deformations known as the ‘normal modes’ that represent the major modes of flexibility of the biomolecule and which have been shown to correspond to protein global motions observed over microsecond or millisecond timescales by atomistic simulation (Gur *et al.*, 2013 ▶). Given the approximations required to produce such a simplified potential function, ENM and GNM frequently also use a coarse-grained protein representation in which a single bead represents each C^α^ atom, and continuum methods based on FEA have also been employed (Bathe, 2008 ▶). The value of these models lies in their simplicity: to calculate the normal modes of a protein of interest a user simply needs to upload the PDB file (for example to the *elNémo* webserver; Suhre & Sanejouand, 2004 ▶) and download the results.

## 4. Computing infrastructure in 2014 in the UK and beyond   

**Figure 5:**
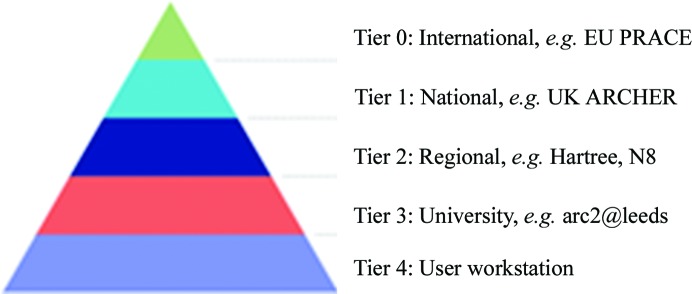
The UK e-infrastructure pyramid.

Just as optical microscopy faces physical limitations on the size and the resolution of the observations that it can make, the ‘computational microscope’ of biomolecular simulation is not infinitely powerful, as even the most sophisticated supercomputers cannot provide unlimited computational power. Indeed, in spite of rapid technological and algorithmic improvements, it is unlikely that supply will meet demand in the near future. However, while the most ambitious bio­molecular simulations make use of high-end computing, many simulations only require modest resources. The provision of computing in the UK and elsewhere, the so-called ‘e-infrastructure’, can be represented as a pyramid, as shown in Fig. 5 ▶. Examples of provision at each of these levels, of how to obtain access and example suitable calculations have been provided in Table 1 ▶.

ARCHER (Academic Research Computing High End Resource; http://www.archer.ac.uk) is the current UK National High Performance Computing Facility, physically located in Edinburgh and operated by EPCC, The University of Edinburgh. The funding for ARCHER is provided through the UK Research Councils, managed by EPSRC. At the time of writing, ARCHER, a CRAY XC30 comprising 3008 nodes, is rated 19 on the list of the worlds’ fastest supercomputers (http://www.top500.org). Such large-scale computers are required to perform leading-edge scientific and engineering simulations that are not feasible on smaller-scale computers. Each ARCHER node can be thought of as analogous to a high-end consumer PC, since it contains commodity processor and memory chips. Each node contains two 12-core Intel Ivy-Bridge processors and 64 GB of memory (with a few nodes featuring larger amounts of memory). Therefore, ARCHER features 72 192 cores and approximately 200 TB of memory in total.

The ARCHER infrastructure is specialized in order to effectively combine multiple processor and memory components into a high-performing large-scale system. The nodes are very densely packed onto compute blades, which slot into cabinets. The servers, plus additional infrastructure (the ARCHER switch room is shown in Supplementary Fig. S1, bottom right), provide the necessary power to the nodes, whilst performing the required cooling through the use of flowing water, as shown in Supplementary Fig. S1 (bottom left). For the nodes to effectively work together on single large-scale problems, they must be able to communicate with each other, and this is facilitated through the availability of the state-of-the-art CRAY Aries high-performance interconnect, as shown in Supplementary Fig. S1 (top right). Large-scale applications also typically require manipulation of large data sets, and ARCHER provides a Lustre parallel file system that allows the efficient reading and writing of application data. ARCHER is co-located with and closely coupled to the Research Data Facility (http://www.epcc.ed.ac.uk/facilities/uk-research-data-facility), which provides a large long-term data-storage area for UK researchers. ARCHER provides a sophisticated and comprehensive software environment, including pre-installed applications and development tools, to help enable researchers to get the best out of the service. Finally, ARCHER also offers support services, ranging from a helpdesk facility to long-term scientific software development programmes. The High End Consortium (HEC) for Bio­molecular Simulation (http://www.hecbiosim.ac.uk) will be providing both supercomputer time and expertise to the community until Autumn 2018. Other routes of access include the EPSRC; supercomputing time can be included on BBSRC responsive mode grants.

For calculations that do not require the full capacity offered by ARCHER, but which nevertheless require specialist supercomputing resources, the UK e-intrastructure provides a number of regional supercomputing centres. The Hartree Centre was opened in February 2013 at the STFC Daresbury Laboratory. There is a particular focus on delivering solutions to industry, but academic/industrial collaborations and infrastructure projects are also supported. Other regional facilities include the N8 supercomputer, Polaris (shown in Supplementary Fig. S1, top left), which is openly available to any researcher at an N8 university (the N8 is a collaborative structure between eight of the northern UK universities), and in the south the Centre for Innovation provides access to conventional HPC computing (IRIDIS) and the EMERALD GPU cluster. Pioneering biomolecular simulators have invested in local GPU clusters, which are generally comparable in price to a multiprocessor workstation, but which at the time of writing can run an MD calculation four times faster than a typical 16-processor workstation using the *AMBER*12 software package. While some established codes (such as *AMBER*; Le Grand *et al.*, 2013 ▶) already have specialized versions that run on GPU technology, much useful software has not yet been adapted and so will not run, or will run only with reduced functionality. Using multiple GPUs for a single calculation may provide only a modest (∼20%) speed-up (the speed-up is very dependent on application and system size); however, the use of a facility such as EMERALD enables many replica simulations to be run, which massively improves conformational sampling (Woods *et al.*, 2013 ▶).

Most useful day-to-day molecular modelling, however, is achieved with conventional, smaller-scale local resources. For example, an eight-processor workstation is capable of performing around 10 ns of modelling a day using *GROMACS* for a solvated protein such as ubiquitin, which contains around 70 residues. This is sufficient, for example, to check the stability of a recently solved biomolecular X-ray or NMR structure, which can provide a basic ‘sanity check’ before the atomic coordinates are deposited. With such a simulation it is possible to identify particularly flexible residues within the protein or to obtain a quick assessment of the stability of a binding pose of a docked ligand. The persistence of key intramolecular or intermolecular contacts can be investigated at room temperature and in the absence of crystal-packing contacts. It can also be used to investigate the possibility that technical details of the experimental procedure, such as the inclusion of His tags for purification or buffer conditions (Majorek *et al.*, 2014 ▶), might possibly influence the outcome of the experiment. Most importantly, molecular modelling and simulation gives researchers the opportunity to visualize their system of interest, which can rapidly change perceptions in surprising and invaluable ways.

## 5. Future perspectives   

Simulations have comparable advantages and caveats to the other experimental techniques presented, and should not be regarded as any less valid so long as they are used appropriately and the corresponding limitations are clearly stated. To conclude, we argue that the biomolecular sciences need to embrace computer simulation as a useful technique for model building and hypothesis testing, especially given the vast quantities of biomolecular data that are being generated. Most insight will be obtained by combining all available biophysical methods to address a single biological problem, and computer simulation can make a valid and valuable contribution. We note that the original longer title for this paper ‘*A Perspective on Computer Simulation as a Biophysical Technique*’ was shortened at the insistence of an anonymous referee, who stated ‘there are insufficient data in the paper to substantiate the connotation of simulation as a biophysical technique’. We leave it to the reader to form their own opinion.

## Supplementary Material

Supplementary Movie S1. DOI: 10.1107/S1399004714026777/ba5225sup1.mpg

Supplementary Movie S2.. DOI: 10.1107/S1399004714026777/ba5225sup2.mpg

Supplementary Movie S3.. DOI: 10.1107/S1399004714026777/ba5225sup3.mpg

Supplementary Movie S4.. DOI: 10.1107/S1399004714026777/ba5225sup4.mov

Supplementary Movie S5.. DOI: 10.1107/S1399004714026777/ba5225sup5.mpg

Supplementary Figure S1 and captions for Supplementary Movies.. DOI: 10.1107/S1399004714026777/ba5225sup6.pdf

## Figures and Tables

**Table 1:** UK supercomputing provision, modes of access and typical suitable calculations for each tier

	Access	QM	MD
ARCHER	http://www.hecbiosim.ac.uk	250 cores, DFT of enzyme with 2500 atoms (10kDa). One geometry optimization requires 200h	512 cores, MD of 256bp DNA circle in water (150kDa), 4 million atoms, up to 50ns
N8	http://www.n8hpc.org.uk	As for ARCHER	64 cores, MD of 100bp DNA circle in water (50kDa), 500000 atoms, up to 100ns
Hartree	hartree@stfc.ac.uk		
EMERALD (GPU) and Iridis	support@einfrastructuresouth.ac.uk		
Local	N/A	High-level QM is normally memory-limited so it is advantageous to own a few local large nodes	16 processors, MD of 20bp DNA in water (10kDa), 35000 atoms, up to 1s
