# Supplementary material for: In pursuit of an accurate spatial and temporal model of biomolecules at the atomistic level: a perspective on computer simulation
Source: Acta Crystallogr D Biol Crystallogr. 2015 Jan 1;71(Pt 1):162–72. doi: 10.1107/S1399004714026777 (PMC4304696; doi:10.1107/S1399004714026777)
Supplement: Supplementary file 6 [file d-71-00162-sup6.pdf]

BIOLOGICAL
CRYSTALLOGRAPHY

Volume 71 (2015)

Supporting information for article:

In pursuit of an accurate spatial and temporal model of biomolecules at the atomistic level: a perspective on computer simulation

Alan Gray, Oliver G. Harlen, Sarah A. Harris, Syma Khalid, Yuk Ming Leung, Richard Lonsdale, Adrian J. Mulholland, Arwen R. Pearson, Daniel J. Read and Robin A. Richardson

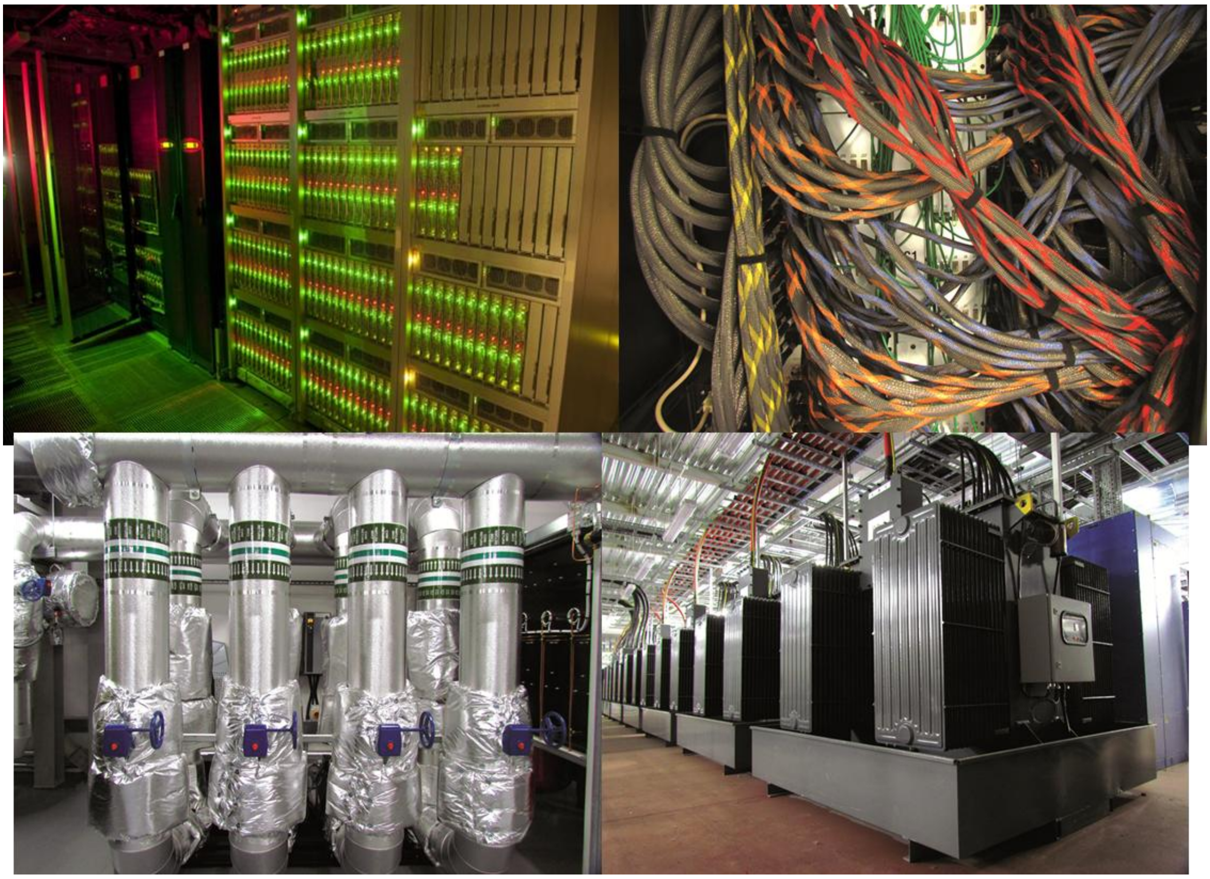

Figure S1 UK supercomputing infrastructure. Top left: The N8 Polaris supercomputer at Leeds. Bottom left: ARCHER's water cooling system. Top right: The ARIES interconnects on ARCHER. Bottom right: ARCHER high voltage switch room.

Movies S1 and S2: QM/MM calculation of the optimal (low energy) pathways for the oxidation of S-warfarin (aromatic hydroxylation) and S-ibuprofen (aliphatic hydroxylation) in the human drug-metabolising cytochrome P450 2C9 (Lonsdale et al., 2013). Detailed insight from reactions such as these is important for understanding the metabolic fate of drugs, especially where formation of toxic metabolites is possible. Both reactions were modelled with QM/MM (using the QoMMMa program (Harvey, 2004)) using multiple starting structures taken from atomistic MD simulations (performed using CHARMM and the CHARMM27 forcefield).

Movie S3: Atomistic molecular dynamics simulation of the KorA protein-DNA complex. The 100ns trajectory has been smoothed to remove high frequency motions.

Movie S4: The movie shows a coarse-grained simulation (using the MARTINI force field within GROMACS 4) of the transmembrane domain of the Fukutin protein. The transmembrane domains of two fukutin proteins (cyan) are embedded with a phospholipid bilayer (head group regions of the lipids are shown in purple). The TXXSS domain of the protein (orange) plays a key role in dimerisation. The proteins are seen approaching each other and then dimerising within microsecond timescale simulations. After the dimerisation event, the peptides remain together for the duration of the 2μs simulations. Two images of each protein are shown through periodic boundary conditions.

Movie S5: The self-assembly of DPPC lipids around OmpA, an outer membrane protein from *E. coli*. The lipid tail particles are cyan, and the head groups particles are blue, gold and green. Water has been omitted for clarity. The initially randomly arranged lipids self-assemble to form a bilayer via the 'stalk' mechanism.

Movie S6: FFEA trajectories of the *M. sexta* V-ATPase, *T. thermophilus* A-ATPase and *S. cerevisiae* V-ATPase molecular motors (Richardson et al, 2014).
